# 

**Published:** 1911-11-25

**Authors:** 


### BOOKS RECEIVED.

HODDER AND STOUGHTON.

"Infectious Diseases and their Preventitive Treatment." ByBy E. C. Seaton.

SCIENTIFIC PRESS, LTD.

"Model Answers to Questions set by the Medico-Psychological." By H. MacPhail.

"The Nurses' Materia Medica." By Herbert French, M.D.

### Buildings Contemplated and in Progress.

Attleborough (Norfolk).—For the erection of the Wayland Infirmary seventeen tenders have been received, varying from £13,996 to £15,521.

Carlisle.—Messrs. J. Burnet and Son, F.R.I.B.A., of Glasgow, have completed the extension and rearrangement of the Cumberland Infirmary at Carlisle at a cost of some £30,000. The work comprises two additional wards, nurses' home, kitchen block and steam laundry, ophthalmic and *x*-ray department, electro-therapeutic rooms, a small chapel, and surgical ward for 12 and children's ward for 24 beds.

Coventry.—For the extension of the Coventry and Warwickshire Hospital a tender of £21,573 is accepted. The successful firm is a local one. To maintain enthusiasm at the annual meeting in December, lantern-slides illustrating the architect's design (Mr. F. J. Cox) will be shown.

Gateshead.—Cottage Homes at Shotley Bridge for the Gateshead Guardians are now approaching completion, and the sanatorium for 64 convalescents is well on the way also. A three-storied hospital to accommodate 300 chronic cases is in progress; the total cost will be about £60,000.

Johannesburg, South Africa.—The accommodation of the Johannesburg Hospital is inadequate to the demand, and extensive additions are projected in consequence. The extension is to cost about £40,000, including two new pavilions and a casualty ward.

London.—A new hospital for paying patients in Vincent Square is projected. Mr. E. Hazell has prepared plans for a three-floored building to accommodate 40 patients. Sir Victor Horsley is on the honorary advisory committee, and considers the scheme both from a medical and an ethical point of view excellent, and it also appears to be on a sound commercial basis.

Winsley (Wilts).—Improvements and extensions are projected at the Counties Sanatorium at Winsley. The cost mentioned is between £8,000 and £9,000.

### Appointments.

Dr. Alfred Bernstein has been appointed Resident Medical Officer at Brompton Hospital for Diseases of the Chest in the room of Dr. A. S. McNalty, who has resigned.

Mr. Hugh T. Campkin has been appointed Dental Surgeon to the Kensington and Fulham General Hospital.

Mr. John Stretton Sloane has been appointed Honorary Surgeon to the Leicester Infirmary on the retirement of Mr. Claude Douglas. Mr. Sloane qualified M.R.C.S., L.R.C.P., in 1893, and was admitted a Fellow of the Royal College of Surgeons in 1895. He graduated B.Sc. London in 1890, M.B. in 1894, B.S. (with first-class honours in surgery) in 1895, and Master of Surgery in 1898. His appointments have included House Surgeon at St. Bartholomew's Hospital, and subsequently Assistant Demonstrator of Anatomy for three years, Chemical Assistant in Surgery, Belgrave Hospital for Children, and Hon. Assistant Surgeon, Metropolitan Hospital, London. Mr. Sloane has been Hon. Assistant Surgeon to the Leicester Infirmary since 1901.

Dr. Harrison Orton has been appointed honorary radiographer to the Mount Vernon Hospital for Consumption in succession to Dr. Pirie, resigned.

Mr. Douglas R. C. Shepherd, M.B., B.S., has been appointed to fill the vacancy in the office of Resident Medical Officer at the Middlesex Hospital created by the election of Mr. Alfred E. Johnson, F.R.C.S., to the honorary staff.

Mr. Walter Lacy Allcroft has joined the Board of Management of the Royal Hospital for Incurables, Putney Heath, filling the vacancy caused by the death of the late Mr. Herbert John Allcroft.

### The Week's Novelties.

### A SALE OF RUBBER GOODS.

At the offices, 37 Queen Victoria Street, E.C., of Messrs. Anderson, Anderson, and Anderson, a clearance sale of their rubber goods is announced. Institutional managers in search of cheap rates for the varied kinds of rubber goods now demanded in all hospitals would do well to obtain a catalogue, and see the prices offered for waterproofs, sheetings, surgeons' gloves, nursing aprons, etc. Carriage will be paid on all parcels of 20s. and over. The West-End branch of the firm's offices is at 58-59 Charing Cross, S.W.

### Florence Nightingale Memorial.

We have received the following contributions since those acknowledged last week; they are for a statue unless otherwise indicated: The Royal Southern Hospital, Liverpool, per Mr. Allen Naldrett, Superintendent and Secretary, £26 6s. (viz. Mr. Thomas Woodsend, £5; Mr. John G. Rodger, J.P., £2 2s.; Mr. E. H. Cookson, J.P., £2; Mr. J. B. Fortune, £1 10s.; Dr. William Alexander, £1 1s.; Dr. Moore Alexander, £1 1s.; Sir William B. Bowring, Bart., £1 1s.; Mr. T. Rowland Hughes, £1 1s.; Mr. R. Cyril Lockett, £1 1s.; Mr. Allen Naldrett, £1 1s.; Mr. Henry Pattinson, £1 1s.; Alderman William Radcliffe, J.P., £1 1s.; Mr. John Rankin, £1 1s.; Dr. William Carter, £1; Mr. D. Douglas Crawford, £1; Mr. Robert Jones, £1; Mr. Percy Rimmer, £1; Dr. J. Lloyd Roberts, £1; Mr. Lyon H. Maxwell, 10s.; Mr. A. Allan Paton, 10s.; Mr. John A. Tinne, 5s.); Miss M. L. Tindal (for annuities for nurses), £5 5s.; A Pension Fund Nurse, 2s. 6d. Total, £31 13s. 6d.

Received by Mr. G. Q. Roberts, Hon. Sec.: Mrs. F. C. Woodhouse, £5 5s.; Mrs. Lyell, £5; A. T. Miller, Esq., £5; Mrs. A. Hickman Morgan, £5; Vice-Admiral Northland, £5; Sir Edward Clouston, Bart., £5; Mrs. Wallis Toller, £3 3s.; the Lord Bishop of Oxford, £3; Reginald Smith, Esq., £2 2s.; Mrs. Boyle, £2 2s.; G. T. Pilcher, Esq., £2 2s.; Herbert E. Cobb., Esq., £2 2s.; C. S. Wallace, Esq., £2 2s.; R. Sharpley, Esq., £2 2s.; "E. A. M.," £2; Mrs. Charles Parbury, £2; Mrs. Kenneth Clark, £2; Nursing Staff of Royal Sussex County Hospital, Brighton, £1 15s. 6d.; Collection at Isolation Hospital, Gateshead, £1 10s.; Past and Present Nurses of Tyrone County Hospital, Omagh, £1 8s.; Mrs. A. E. Abrahams, £1 1s.; Miss Winifred Bullen, £1 1s.; Mrs. Lloyd Thomas, £1 1s.; H. E. Medlicott, Esq., £1 1s.; Mrs. Bompas, £1 1s.; Mrs. Davies, £1 1s.; "A Soldier," £1 1s.; Mrs. Lillingston, £1 1s.; Sir Sandford Fleming, K.C.M.G., £1 1s.; Dr. de Havilland Hall, £1 1s.; R. C. Abbott, Esq., £1 1s.; Col. R. H. P. Doran, £1; the Dowager-Countess of Derby, £1; Mrs. Arthur Loring, £1; Mr. and Mrs. R. C. Rogers, £1. Total, £75 4s. 6d.

Received by Australian Trained Nurses' Association, £42 11s.

Further contributions may be sent to the Hon. Secretary Florence Nightingale Memorial, St. Thomas's Hospital, Albert Embankment, London, S.E., or to the Editor, The Hospital, 29 Southampton Street, Strand, London, W.C.

### Gifts.

Mr. David Fenby has left £500 to each of the following institutions: The Royal Infirmary, Sheffield; the Sheffield Royal Hospital; the Jessop Home for Women; and the Crookes Orphan Homes.

Bradford Royal Infirmary.—Mr. John Holmes, of Dudley Hill, Bradford, has given a donation of £500 to endow a cot in the Bradford Royal Infirmary; and £500 has been received from Mr. Joseph Craven, of Thornton, to the general funds of the Royal Infirmary. The Lord Mayor of Bradford has received from the Mayor of Pudsey two cheques—one for £122 2s. 5d., to the general funds of the Bradford Royal Infirmary, and the other for £75 0s. 9d., to the Bradford Eye and Ear Hospital. These amounts are part of the sum collected in Pudsey for the King Edward Memorial Fund.

Miss M. Haslam Marsden, of Birkdale, has left £100 to the Bolton Infirmary.

Mr. James Young, of Sherborne, Dorset, has left £100 to the Yeatman Hospital, Sherborne.

Miss Betsy Crossley has bequeathed £1,000 to the Halifax Royal Infirmary.

Colonel Thomas Simpson has bequeathed £500 to the Armagh County Infirmary.

Mr. William Hampton, of Putney, has left £500 to the Bolingbroke Hospital, Wandsworth.

Mr. R. Ayrton England, of Cotehill, Cumberland, has left £750 to the Bingley Cottage Hospital.

Mr. Max Rosenheim has bequeathed £100 to the Hampstead General Hospital and £100 to the London Hospital.

Mrs. E. Durrant Russell, of Darlington, has bequeathed £100 to the Gravesend Hospital and £100 to the London Hospital.

Mr. Robert Morton has left, subject to the life interest of his wife, the sum of £4,000 to the National Society for the Employment of Epileptics.

Miss Elizabeth Smith, of Birkdale, has left £200 to the North of England Sanatorium, Southport, and £100 to Dr. Barnardo's Home for Cripples, Birkdale.

The Worshipful Company of Salters has sent its annual subscription of twenty guineas to the Treasurer of the Royal Hospital for Incurables, Putney Heath.

Under the will of the Rev. Canon Robinson Duckworth the Westminster Hospital, the Hospital for Incurable Children, Hampstead, and the Hostel of St. Luke, Fitzroy Square, each receive £100.

The Treasurers of the Middlesex Hospital have received a further donation of £100 from Mr. C. H. Berners, and £100 in memory of Theresa de G. Miller, towards the maintenance of the Cancer Research Department.

Miss E. M. Gent has bequeathed £1,000 to the Seaford Convalescent Hospital, and the same amount to the Governors of Addenbrooke's Hospital, Cambridge, and to the Eastern Counties Asylum for Idiots, Colchester.

Mr. Alfred Sims, of Herne Bay, has left £500 each to Dr. Barnardo's Homes and the National Benevolent Institution, and £200 to the Dispensary, Wilkinson Street, Clapham Road, and £200 to the Queen Victoria Hospital.

Mr. L. Haslop Williamson, of Buxton, has bequeathed on his wife's decease £400 each to the Manchester Royal Infirmary and Dispensary, the Manchester Northern Hospital for Women and Children, the St. Mary's Hospital for Women and Children, and the General Hospital for Sick Children at Pendlebury.

The late Countess Dowager of Seafield has bequeathed £1,000 to the Infirmary, Inverness; £1,000 to the Chalmers Hospital, Banff; and £18,000 to the trustees of the Ian Charles Cottage Hospital at Grantown, the interest on which it is to be applied to the upkeep of the hospital, any surplus to be accumulated to meet expenses in the future.

Mr. Daniel Myers has bequeathed £1,000 to the London Hospital to endow a "Sophia Myers" bed in memory of his late wife; £500 each to Guy's Hospital, the Jews' Hospital and Orphan Asylum, West Norwood; £300 each to the Home and Hospital for Jewish Incurables, Tottenham, the Metropolitan Hospital, Kingsland Road, the North-Eastern Hospital for Children, the East London Hospital for Children, the Hospital for Sick Children, Great Ormond Street, the City of London Hospital for Diseases of the Chest, the Cancer Hospital, the Royal Free Hospital, Gray's Inn Road, the Middlesex Hospital, University College Hospital, St. Mary's Hospital, Paddington, and the Poplar Hospital.

